# Cochlear Implantation in the United Arab Emirates: Otolaryngologists’ Knowledge, Attitudes, and Practices

**DOI:** 10.3390/audiolres15020044

**Published:** 2025-04-21

**Authors:** Muhammed Ayas, Ahmad Al Amadi, Ahmad Al Shamsi, Jameel Muzaffar, Manohar Bance

**Affiliations:** 1 College of Health Sciences, University of Sharjah, Sharjah P.O. Box 27272, United Arab Emirates; 2 Department of Clinical Neurosciences, University of Cambridge, Cambridge CB2 0QQ, UK; 3 Cambridge Hearing Group, University of Cambridge, Cambridge CB2 1TN, UK; 4 Research Institute of Medical and Health Sciences (RIMHS), University of Sharjah, Sharjah P.O. Box 27272, United Arab Emirates; 5 Advance Hearing and Balance Centre, Dubai P.O. Box 6867, United Arab Emirates; 6 Shaikh Shakhbout Medical City, Abu Dhabi P.O. Box 7762, United Arab Emirates; 7 Department of ENT, University Hospitals Birmingham NHS Foundation Trust, Birmingham B15 2GW, UK; 8 Department of Applied Health Sciences, University of Birmingham, Birmingham B15 2TT, UK; 9 Department of ENT, Cambridge University Hospitals NHS Foundation Trust, Cambridge CB2 0QQ, UK

**Keywords:** cochlear implants, otolaryngologists, knowledge, attitudes, practices, hearing loss

## Abstract

**Background:** Cochlear implants (CIs) are the gold standard intervention for severe-to-profound sensorineural hearing loss. Their success depends not only on technological advancements but also on the knowledge, attitudes, and practices (KAP) of otolaryngologists responsible for patient selection, counselling, and postoperative management. **Objective:** This study aimed to evaluate the KAP of otolaryngologists in the UAE towards CIs, identify gaps in training and decision-making, and assess adherence to global CI protocols. **Methods:** A cross-sectional study was conducted using a self-administered online questionnaire distributed to otolaryngologists across public and private healthcare institutions in the UAE. The survey assessed demographics, clinical knowledge, attitudes towards CIs, and clinical practices. Descriptive and inferential statistical analyses were performed to assess the relationships among knowledge levels, referral frequency, and institutional factors. **Results:** A total of 31 otolaryngologists participated. While 74% demonstrated moderate-to-high knowledge of CIs, 39% had low awareness of national guidelines. Most (61%) strongly supported CI integration into treatment protocols, but financial and institutional barriers were frequently cited as challenges. Knowledge levels significantly correlated with referral frequency (*p* < 0.001), indicating a gap between awareness and practice. **Conclusions:** This is the first study in the UAE assessing otolaryngologists’ KAP regarding CIs. Despite favourable attitudes, limited guideline awareness, financial barriers, and inconsistent multidisciplinary collaboration remain challenges. Targeted clinician education, standardised CI guidelines aligned with international benchmarks, and improved funding mechanisms are essential to enhance CI accessibility and optimise patient outcomes in the region.

## 1. Introduction

Cochlear implants (CIs) are a transformative intervention for individuals with severe-to-profound sensorineural hearing loss (SNHL). By bypassing damaged inner ear structures and directly stimulating the auditory nerve, these devices restore the perception of sound, significantly enhancing communication, social integration, and overall quality of life [1]. The success of CIs depends not only on technological advancements but also on the clinical expertise of otolaryngologists in selecting candidates, performing surgeries, and managing postoperative care as a part of a multidisciplinary team [2,3].

Achieving optimal outcomes requires the integration of technology with high-quality clinical care. Detailed candidacy evaluation, precise surgical planning, and comprehensive postoperative management are essential to translate the potential of CIs into real-world benefits [4,5]. In rapidly evolving healthcare environments in the Middle East, particularly in the United Arab Emirates (UAE), the implementation of CI practices faces unique challenges. Several factors contribute to heterogeneous CI practices, including variability in clinician training, differences in exposure to international CI protocols, and disparities in resource allocation [6]. These disparities underscore the need for a critical evaluation of clinical practices against evidence-based guidelines and the identification of opportunities for improved training and resource allocation.

While the technological advancements in CIs are well documented in the existing literature [7,8], there is a limited focus on the critical role of otolaryngologists’ knowledge, attitudes, and practices (KAP) in shaping CI outcomes. Studies from developed countries suggest that well-informed and proactive clinicians significantly enhance patient experiences, improve device utilisation, and contribute to higher rehabilitation success rates [9,10]. In contrast, research from developing nations highlights challenges such as limited awareness, clinician hesitancy, and infrastructural barriers, all of which hinder CI accessibility and success [11,12].

Beyond speech perception and patient-centred outcomes, the role of clinicians is integral to CI success. The decision to recommend a CI, the counselling provided to patients, and the postoperative management all hinge on the expertise and attitudes of otolaryngologists along with other professionals [13]. Thus, understanding clinicians’ knowledge, perceptions of CIs, and alignment with best evidence is essential for improving CI outcomes at both individual and systemic levels.

Despite the increasing adoption of CIs in the UAE, there is limited data on the perspectives of otolaryngologists regarding their implementation. Understanding their KAP is critical for optimising CI programmes. This study aims to bridge this gap by systematically evaluating the KAP of otolaryngologists in the UAE toward CIs, with a focus on identifying gaps in training, decision-making processes, and adherence to global CI protocols. By exploring how clinician perspectives influence clinical decisions and patient outcomes, we seek to create a more informed, engaged, and effective healthcare ecosystem for individuals who are expected to benefit from CI.

## 2. Materials and Methods

### 2.1. Study Design

A cross-sectional study design was employed to assess the KAP of otolaryngologists regarding CIs in the UAE. Data were collected through a bespoke, self-administered online questionnaire designed after an extensive review of the relevant literature and consultations with both regional and international experts. The data collection period extended from May 2024 to January 2025.

### 2.2. Ethical Considerations

The study was conducted in accordance with the ethical standards outlined in the Declaration of Helsinki. Electronic informed consent was obtained from all participants prior to data collection. The study was approved by the Research and Ethics Committee of University of Sharjah [REC-24-03-26-9411-F].

### 2.3. Participants and Recruitment

Eligible participants included practicing otolaryngologists in both public and private healthcare settings across the UAE. Recruitment was facilitated through professional networks, social media platforms, and ENT societies. The survey invitation was shared with the aim of reaching all eligible clinicians across the Emirates. Due to the nature of the survey, the exact number of recipients could not be verified. Given an estimated target population of more than 100 otolaryngologists in the UAE, the study aimed for a minimum sample size of approximately 41 to 48 respondents to achieve adequate representativeness, depending on the total population size. However, 31 responses were obtained, which provided an 80% chance of detecting correlations of ±0.50 at *p* ≤ 0.05 based on Cohen’s power analysis equation [14].

To account for potential confounding factors, demographic and professional variables were collected, including years of experience, prior CI-related training, practice setting, and institutional CI policies. These factors were considered in the analysis to assess their potential impact on KAP. Since recruitment was conducted via professional networks and social media, there may be a chance of overrepresentation of clinicians with existing interest in CIs, potentially influencing the findings.

### 2.4. Questionnaire Development

The questionnaire was developed to capture comprehensive data on demographic characteristics, clinical knowledge, attitudes toward CI, and practical aspects of CI management. It featured multiple-choice items, open-text responses, and visual analogue scales (VAS) to capture detailed insights. The instrument included 28 questions and was structured in four sections (Supplementary Material).

1. Demographics: Five questions addressing age, sex, practising location, years of experience, and subspecialty.
2. Knowledge: Seven questions assessing familiarity with CI procedures, guidelines, and related clinical information.
3. Attitudes: Seven questions assessing perceptions of CI effectiveness, benefits, and barriers.
4. Clinical Practices: Nine questions exploring methods of patient evaluation, surgical planning, and postoperative management.

### 2.5. Pilot Testing and Validation of Questionnaire

A panel comprising two experienced otolaryngologists and two experienced audiologists reviewed the initial version of the questionnaire, resulting in refinements. Subsequently, a pilot test was conducted with five otolaryngologists to evaluate clarity and feasibility, which led to minor modifications. Internal consistency was measured using Cronbach’s alpha values ranging from 0.78 to 0.85, indicating good validity. Reliability was assessed by re-administering the questionnaire to the same group, yielding Kappa scores between 0.66 and 0.74 for the categorical items in the questionnaire, which indicates substantial agreement [15]. Data obtained during the pilot phase were not included in the final analysis.

### 2.6. Data Analysis

Collected data were analysed using descriptive statistics (means, standard deviations, frequencies, and percentages) to summarise participant responses. Inferential statistical tests, correlation analyses, were employed to examine the associations between variables. Statistical significance was set at *p* ≤ 0.05, and analyses were performed using Statistical Package for the Social Sciences (SPSS) Version 30.

## 3. Results

### 3.1. Participant Characteristics

A total of 31 otolaryngologists participated in the study. The majority were male (68%), with female respondents constituting 32%. Most participants (14/31, 45%) were aged between 45 and 54 years, followed by 7/31 (23%) in the 35–44 age group. Since respondents could select multiple subspecialties, the counts reflect the number of clinicians indicating expertise in each area rather than the total number of participants. Among the respondents, 13 (42%) were general otolaryngology, followed by otology/neurotology (10, 32%), rhinology (9, 29%), laryngology (6, 19%), head and neck surgery (5, 16%), paediatric otolaryngology (4, 13%), facial plastic and reconstructive surgery (3, 10%), and sleep medicine (3, 10%). Regarding professional experience, 39% had 11–20 years of practice, while 36% had over 20 years of experience and 26% had less than 10 years of experience. Table 1 summarises the participants’ characteristics.

### 3.2. Knowledge of CI

**Figure 1:**
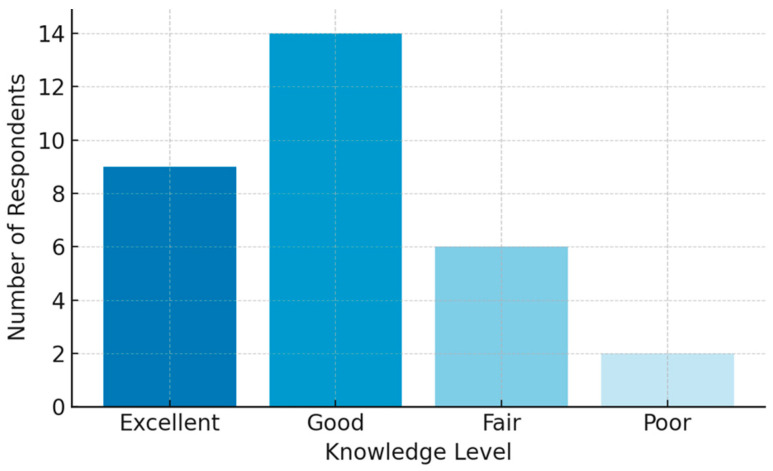
Self-reported knowledge of CI procedures.

Participants rated their CI knowledge on a four-point scale (Excellent, Good, Fair, Poor) with **29%** rating their knowledge as excellent (95% CI: 13.1–45.0%), **45%** as good (95% CI: 27.6–62.7%), **19%** as fair (95% CI: 5.4–33.3%), and **6%** as poor (95% CI: 0.0–15.1%) (Figure 1). While **77%** correctly identified that CIs function by directly stimulating the auditory nerve, only **48%** correctly recognised both congenital deafness and acquired SNHL as primary indications, suggesting a gap in knowledge of CI candidacy. Potential complications associated with CI surgery were most identified as infection, device failure, and facial nerve damage (**42%**).

### 3.3. Attitudes Towards CIs

**Figure 2:**
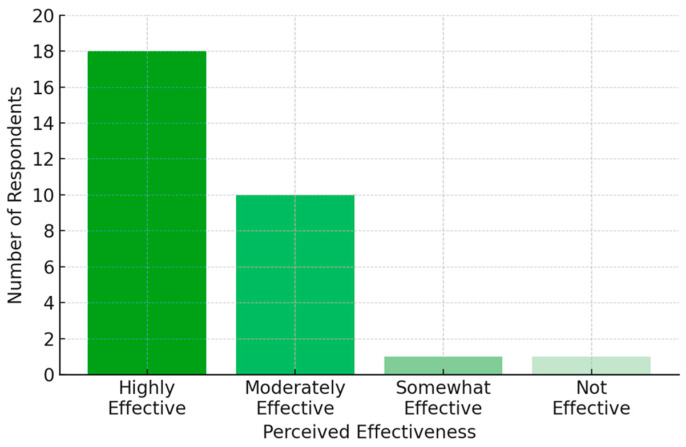
Attitudes toward CI effectiveness.

Most respondents (61%) perceived CIs as highly effective, while 32% considered them moderately effective. A significant proportion (84%) believed that CIs provide superior outcomes compared to hearing aids (Figure 2). Opinions on potential cultural or societal barriers were divided, with 36% acknowledging such barriers, while 29% disagreed. Almost all participants (94%) believed that CIs are essential for improving the quality of life of patients with severe-to-profound hearing loss. Additionally, 61% strongly agreed that CIs should be integrated into routine clinical practice for hearing loss. Regarding the preferred implantation strategy, 90% of participants supported bilateral CIs for children, whereas for adults, 61% favoured bilateral implantation. Awareness of national CI guidelines was limited, with 39% reporting low awareness levels. In terms of referral frequency, 42% recommended CIs occasionally, while an equal proportion recommended it very frequently.

### 3.4. Clinical Practices and Decision-Making

**Figure 3:**
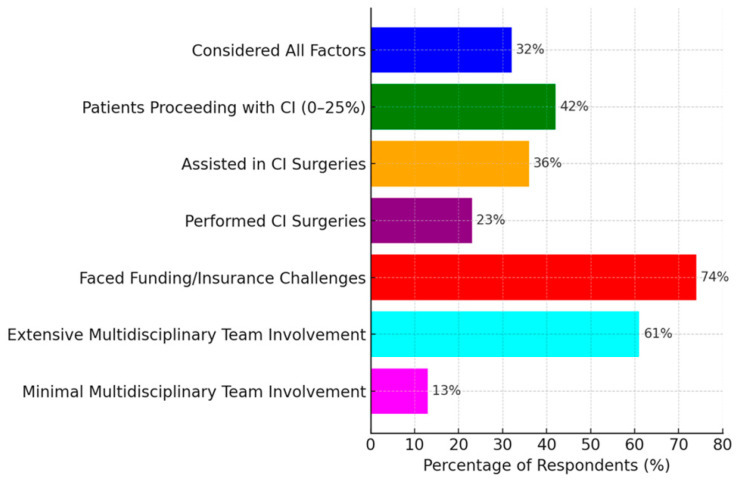
Clinical practices and decision-making in CI recommendation.

Key factors influencing CI recommendations included the severity of hearing loss, age, financial considerations, and the presence of comorbidities, with 32% considering all these factors in combination. Only 42% of respondents reported that 0–25% of their eligible patients proceeded with implantation (Figure 3). Regarding surgical experience, 36% had assisted in CI surgeries, and 23% had performed them. Notably, 74% of participants reported encountering funding or insurance challenges for CI procedures, with financial constraints likely acting as a major barrier to wider CI adoption. Multidisciplinary team involvement was extensive for 61% of respondents, while 13% reported minimal involvement.

### 3.5. Factors Influencing Knowledge, Attitudes, and Practices

Knowledge levels differed significantly based on years of practice (*p* = 0.014), with more experienced practitioners demonstrating higher knowledge scores. However, no significant differences were found in attitudes towards CIs across different regions. Similarly, practice patterns did not vary significantly across subspecialties. This finding is noteworthy, as one might expect certain subspecialties, such as rhinology or facial plastics, to have lower knowledge levels regarding CIs. A possible explanation could be that the knowledge assessment was self-reported, potentially reflecting differences in self-awareness rather than actual knowledge disparities.

### 3.6. Predictors of CI Recommendation and Multidisciplinary Involvement

Higher knowledge and awareness were significantly associated with greater CI recommendation frequency (*p* < 0.001). Higher awareness of national guidelines and better knowledge of CI procedures were associated with a greater likelihood of recommending CIs. Additionally, knowledge and experience were significant predictors of multidisciplinary team involvement (*p* < 0.001), with greater experience and higher knowledge scores correlating with more extensive team-based care.

## 4. Discussion

CIs are a well established intervention for individuals with severe-to-profound hearing loss, yet their successful implementation depends on clinicians’ KAP. This study examined the KAP of otolaryngologists in the UAE, revealing notable variations in awareness of CI eligibility, clinical protocols, and access to post-implantation rehabilitation. While most clinicians acknowledged CIs as effective intervention, gaps in guideline awareness, referral pathways, and interdisciplinary collaboration emerged as key challenges. Additionally, financial and institutional barriers were identified as significant impediments to CI uptake.

### 4.1. Knowledge and Awareness of CIs

Despite relatively high self-reported knowledge levels, the findings of the study suggest opportunities to enhance awareness of national guidelines [16]. Familiarity with national criteria appears to be less robust compared to broader international recommendations, including those from the UK National Institute for Health and Care Excellence (NICE), the US Food and Drug Administration (FDA), and the World Health Organization (WHO). This limited familiarity may lead to inconsistencies in referral patterns, surgical planning, and postoperative care, potentially affecting patient outcomes [12]. While clinicians correctly identified fundamental aspects of CIs, such as their function in stimulating the auditory nerve and key indications, the variability in recognising potential surgical complications, such as infection, device failure, and facial nerve damage, highlights a need for more structured education on perioperative risk management [17].

A lack of standardised training programmes on CI guidelines may contribute to these discrepancies, underscoring the importance of continued medical education (CME) initiatives. Studies have demonstrated that structured training, protocol dissemination, and targeted professional development programmes improve clinician competency and enhance adherence to evidence-based practices [17,18]. Addressing these gaps through well-integrated professional education frameworks could ensure a more consistent approach to CI management, ultimately leading to better patient care and outcomes. Given the low awareness of national CI protocols and variability in clinical practices observed in this study, aligning UAE guidelines with international standards may support more consistent and evidence-based decision-making across the country.

### 4.2. Attitudes Toward CIs and Their Utility

The findings suggest that clinicians largely perceive CIs as effective intervention, with a strong consensus on their superiority over hearing aids in improving speech perception and communication outcomes. This aligns with the existing literature highlighting the significant benefits of CIs in individuals with severe-to-profound hearing loss [19]. Furthermore, there was broad agreement on the essential role of CIs in enhancing patients’ quality of life, reinforcing the need for their continued integration into routine clinical care [19,20].

Despite these positive attitudes, variability was observed in clinicians’ perceptions of cultural and societal barriers to CI adoption. Some clinicians recognised these barriers, while others did not perceive them as significant. This discrepancy may reflect differences in clinical exposure, patient demographics, or regional variations in healthcare accessibility [21]. Interestingly, attitudes toward CIs did not significantly differ across regions, despite expected variations in healthcare accessibility. This finding may indicate that clinician perspectives are shaped more by institutional policies and training exposure than geographic factors. Given that societal acceptance plays a critical role in CI uptake, targeted public awareness campaigns and patient-centred counselling may help address concerns and facilitate informed decision-making.

The need for bilateral CIs was strongly recognised for the paediatric population, in line with recommendations emphasising the benefits of binaural auditory input for auditory and language development. While the benefits of bilateral CIs in children are well established, the variability in clinician support for adult bilateral CIs suggests concerns regarding both cost-effectiveness and patient-specific factors, such as the duration of deafness and rehabilitation challenges. Given the evolving evidence-based data on adult bilateral CIs [22], further professional education on bilateral CI outcomes in adults may help inform practice. The limited awareness of national CI guidelines among UAE otolaryngologists highlights a need for greater dissemination of evidence-based protocols. A lack of clear national guidelines has been associated with variability in referral patterns and clinical decision-making in other regions as well. Strengthening national CI guidelines and ensuring alignment with international standards may improve consistency in clinical practice and patient referrals.

### 4.3. Clinical Practices, Decision-Making, and Barriers to CI Implementation

Despite widespread recognition of CI benefits, a significant gap persists between clinical recommendations and actual patient uptake. Clinicians reported considering multiple factors in their decision-making, including severity of hearing loss, age, financial constraints, and comorbidities [22]. However, the relatively low proportion of eligible patients proceeding with implantation suggests that non-clinical barriers, such as healthcare accessibility, financial limitations, and patient awareness, play a critical role in influencing outcomes [23].

Financial constraints emerged as a key challenge, with many clinicians citing difficulties related to insurance coverage and funding approvals, mirroring trends observed in other healthcare systems [16,24]. Addressing these issues through expanded insurance coverage, streamlined reimbursement processes, and policy-driven solutions may improve CI accessibility. Institutional disparities in CI practices further highlight the need for national clinical guidelines to standardise patient care. While some clinicians reported extensive multidisciplinary collaboration, others indicated limited engagement with audiologists, speech–language pathologists, and rehabilitation specialists, underscoring inconsistencies in interdisciplinary involvement. Given the well-documented benefits of coordinated care, enhancing multidisciplinary collaboration is essential for optimising CI outcomes [25].

Beyond clinical and institutional barriers, patient hesitancy remains a concern. Misconceptions regarding CI maintenance, social stigma, financial burden, and fear of surgery contribute to delays in implantation [13,23]. The social stigma associated with wearing a visible medical device, particularly in cultures where hearing loss is not openly discussed, can also deter potential candidates from pursuing CIs. Unlike government-backed CI programmes in the UK and USA [26], the UAE primarily relies on a mix of insurance and government initiatives. Expanding reimbursement mechanisms and streamlining preauthorization processes could alleviate these financial constraints.

### 4.4. Implications for Policy and Clinical Practice

The findings of this study highlight several key areas for improvement in CI programmes, with implications for both policy and clinical practice. Strengthening national CI guidelines and ensuring alignment with international standards could promote consistency in clinical decision-making and referral patterns. Expanding insurance coverage and financial support mechanisms may also improve accessibility for eligible patients, reducing cost-related barriers.

Addressing knowledge gaps through continued medical education is essential for optimising CI outcomes. Integrating structured CI training into otolaryngology residency programmes and CME courses may enhance guideline adherence. Standardised training in CI surgery, rehabilitation, and evidence-based management could enhance clinician confidence and improve adherence to best practices. Additionally, the variability in multidisciplinary collaboration suggests the need for structured referral pathways that integrate audiologists, speech–language pathologists, and rehabilitation specialists more effectively.

Public awareness campaigns could help address societal and cultural barriers to CI uptake. Patient education and counselling should be prioritised to remove common misconceptions, improving acceptance and adherence to CI rehabilitation protocols.

### 4.5. Limitations

This study has some limitations that should be considered when interpreting the findings. The sample size, while representative, may not capture the full spectrum of CI practices across all healthcare institutions in the UAE. The response rate was 31% and given the relatively low participation, selection bias is possible. Clinicians more engaged with CIs may have been more likely to participate, potentially leading to an overestimation of knowledge and positive attitudes towards CIs. Additionally, the study relied on self-reported data, which introduces potential response bias. Participants may have overestimated their knowledge or adherence to CI guidelines due to social desirability bias. Future studies should incorporate objective assessments, such as clinical case evaluations or knowledge tests, to validate self-reported responses.

Although the survey was circulated through national ENT societies and professional networks to reach all eligible otolaryngologists in the UAE, participation was voluntary and anonymous, limiting our ability to track the exact number of recipients or follow up with non-responders. This likely contributed to the limited sample size. While 57% of participants reported performing or assisting in CI surgery, subgroup comparisons based on CI experience were not conducted due to the small sample size and associated power limitations. Future studies with larger cohorts may allow for meaningful subgroup analyses.

Furthermore, this study primarily focused on otolaryngologists, whereas the perspectives of audiologists, speech–language pathologists, and rehabilitation professionals were not explored in depth. Given that audiologists often play an important role in identifying potential CI candidates and guiding them toward implantation, their perspectives are essential for a more comprehensive understanding of referral patterns and patient decision-making. A multidisciplinary approach is necessary to gain a more comprehensive understanding of CI-related practices. Institutional policies, financial constraints, and healthcare infrastructure were not assessed in detail, yet these factors play a crucial role in determining CI accessibility and patient uptake. Future research should investigate the impact of policy-level and institutional barriers on CI adoption to better inform healthcare decision-making.

### 4.6. Future Research

Future studies exploring the effectiveness of structured clinician training programmes and their impact on CI decision-making and patient counselling would be beneficial. Qualitative research approaches, such as semi-structured interviews, could provide deeper insights into clinician perspectives, patient experiences, and institutional barriers affecting CI uptake. Such methods could explore themes related to clinical decision-making, interdisciplinary collaboration, and real-world challenges in CI implementation. Additionally, examining the role of audiologists and speech–language pathologists in CI referral pathways, patient counselling, and post-implant rehabilitation would be beneficial.

Future studies should examine whether CME courses, simulation-based training, or multidisciplinary workshops improve guideline adherence and referral accuracy. Investigating patient perspectives on CI barriers and facilitators could help develop more patient-centred educational strategies. Further, cost-effectiveness analyses of CI programmes should be conducted to explore potential funding models and identify gaps in insurance reimbursement policies that may limit access for individuals from different socioeconomic backgrounds. Moreover, it is essential to examine how clinician attitudes translate into real-world referral behaviours and patient outcomes, allowing for a more evidence-driven integration of CIs into clinical pathways.

## 5. Conclusions

To our knowledge, this is the first study in the UAE to assess otolaryngologists professionals’ KAP regarding CIs. While attitudes were generally favourable, awareness of national guidelines remained low. Financial barriers and limited multidisciplinary engagement were also identified as key challenges affecting CI uptake. These findings highlight the urgent need for targeted clinician education, policy interventions, and improved institutional frameworks to enhance CI accessibility. Strengthening national CI guidelines aligned with international benchmarks, expanding funding mechanisms, and fostering interprofessional collaboration among otolaryngologists, audiologists, and rehabilitation professionals are essential steps toward optimising CI outcomes in the region.

## Figures and Tables

**Table 1:** Demographic characteristics of participants.

Demographic Characteristic	Count	Percentage
**Gender**		
Female	10	32%
Male	21	68%
**Age**		
25–34	5	16%
35–44	7	23%
45–54	14	45%
55 or above	5	16%
**Practicing Emirate**		
Abu Dhabi	10	32%
Dubai	12	39%
Sharjah	5	16%
Ajman	1	3%
Umm al Quwain	1	3%
Ras al-Khaimah	1	3%
Fujairah	1	3%
**Subspecialty**		
General Otolaryngology	13	42%
Otology/Neurotology	10	32%
Rhinology	9	29%
Laryngology	6	19%
Head and Neck Surgery	5	16%
Paediatric Otolaryngology	4	13%
Facial Plastic and Reconstructive Surgery	3	10%
Sleep Medicine	3	10%
**Years of Experience**		
Less than 5 years	1	3%
5–10 years	7	23%
11–20 years	12	39%
Over 20 years	11	36%

## Data Availability

The datasets used and analysed in the current study can be obtained from the corresponding author, subject to reasonable request.

## Supplementary Materials

The following supporting information can be downloaded at: https://www.mdpi.com/article/10.3390/audiolres15020044/s1, Survey on Knowledge, Attitude, and Practice Regarding Cochlear Implantation among Otolaryngologists in the UAE.

## Abbreviations

The following abbreviations are used in this manuscript:

CIs	Cochlear implants
SNHL	Sensorineural hearing loss
KAP	Knowledge, attitudes, and practices
CME	Continuing Medical Education
