# Supplementary material for: Cochlear Implantation in the United Arab Emirates: Otolaryngologists’ Knowledge, Attitudes, and Practices
Source: Audiol Res. 2025 Apr 21;15(2):44. doi: 10.3390/audiolres15020044 (PMC12024090; doi:10.3390/audiolres15020044)
Supplement: Supplementary file 1 [file audiolres-15-00044-s001.zip › audiolres-3563769-Supplimentary material -KAP ENT CI Questionnaire (1).pdf]

Title: Survey on Knowledge, Attitude, and Practice Regarding Cochlear Implantation among Otolaryngologists in the UAE

1. Gender:
    1. Male
    2. Female

2.Age:
1. Under 25
2. 25-34
3. 35-44
4. 45-54
5. 55 or above

3.Practicing Emirate
1. Abdu Dhabi
2. Dubai
3. Sharjah
4. Ajman
5. Umm al Quwain
6. Ras Al Khaimah
7. Fujairah

4.Subspecialty:(Select all that apply)
1. Rhinology
2. Laryngology
3. Head and Neck Surgery
4. Pediatric Otolaryngology
5. Facial Plastic and Reconstructive Surgery
6. Sleep Medicine
7. Other (please specify): ______
8. Otology/Neurotology

5.Years of Practice:
1. Less than 5 years
2. 5-10 years
3. 11-20 years
4. Over 20 years

Knowledge:

6.How would you rate your knowledge of cochlear implantation procedures?

4.Excellent

3.Good

2.Fair
1.Poor

7.Which of the following conditions are commonly treated with cochlear implants? (Select all that apply)(binary coding)

A. Congenital deafness
B. Acquired sensorineural hearing loss
C. Otosclerosis
D. Presbycusis (age-related hearing loss)

8.How does a cochlear implant function?

0. By amplifying sound waves
1. By stimulating the auditory nerve directly
0. By replacing damaged hair cells in the cochlea
0. By conducting sound vibrations through bone conduction

9.What are the potential complications or risks associated with cochlear implant surgery? (Select all that apply) binary coding)

A. Infection
B. Device failure
C. Damage to facial nerve
D. Loss of residual hearing

10.How confident are you in your ability to interpret audiometric evaluations and determine whether a patient is a suitable candidate for cochlear implantation?

4. Very confident
3. Somewhat confident
2. Neutral
1. Not confident

11.How do you stay updated with the latest advancements and techniques in cochlear implantation?

A. Attendance at conferences/seminars
B. Continuing medical education courses
C. Medical journals/publications
D. Online resources/forums
E. Other (please specify): ______

12.When considering a patient for cochlear implantation, which factors do you believe are important in determining their candidacy? (Select all that apply) binary coding)

A. Severity of hearing loss
B. Speech perception abilities
C. Use of hearing aids with limited benefit
D. Age of the patient

Attitude:

13.How do you perceive the effectiveness of cochlear implants in restoring hearing?

4. Highly effective
3. Moderately effective
2. Somewhat effective
1. Not effective

14.What are your beliefs regarding the benefits of cochlear implantation compared to traditional hearing aids?

4. Cochlear implants provide superior outcomes
3. Cochlear implants and hearing aids have comparable outcomes
2. Hearing aids are more effective than cochlear implants
1. Unsure/No opinion

15.Do you think there are any cultural or societal barriers that may affect the acceptance of cochlear implants among patients in the UAE?

3. Yes
2. No
1. Unsure

16.How do you perceive the role of cochlear implantation in improving the quality of life for patients with severe to profound hearing loss?

4. Essential for enhancing quality of life
3. Beneficial but not essential
2. Neutral
1. Not significant

17.To what extent do you believe that cochlear implantation should be integrated into standard treatment protocols for hearing loss management?

4. Strongly agree
3. Agree
2. Disagree
1. Strongly disagree

18.Which do you think is better for children with hearing loss: having cochlear implants in both ears or just one?

3. Bilateral cochlear implantation (both ears)
2. Unilateral cochlear implantation (one ear)
1. Unsure/No opinion

19.Which do you think is better for adults with hearing loss: having cochlear implants in both ears or just one?

3.Bilateral cochlear implantation (both ears)
2.Unilateral cochlear implantation (one ear)
1.Unsure/No opinion

Practice:

20.How aware are you of the national guidelines or protocols for cochlear implantation in the UAE?

4. Very aware
3. Somewhat aware
2. Not very aware
1. Not aware at all

21.How frequently do you recommend cochlear implantation as a treatment option for eligible patients?

4. Very frequently
3. Occasionally
2. Rarely
1. Never

22.What factors influence your decision to recommend cochlear implantation? (Select all that apply)

Severity of hearing loss
Age of the patient
Presence of comorbidities
Financial considerations

23.What percentage of your patients who are candidates for cochlear implants undergo the procedure?

4. 0-25%
3. 26-50%
2. 51-75%
1. 76-100%

24.Have you had previous experience with performing or assisting in cochlear implantation surgeries or procedures?

4. Yes, I have performed cochlear implantation surgeries.
3. Yes, I have assisted in cochlear implantation surgeries.
2. No, I have not been directly involved in cochlear implantation surgeries.
1. No, I have no experience with cochlear implantation surgeries.

25.Have you encountered any challenges in obtaining funding or insurance coverage for cochlear implantation procedures for your patients?

2.Yes
1.No

26. To what extent do you involve multidisciplinary teams, such as audiologists, speech-language pathologists, and psychologists, in the evaluation and management of patients undergoing cochlear implantation?

4.Extensively
3.Moderately
2.Minimally
1.Not at all

27. Have you received formal training or education on cochlear implantation procedures?

3.Yes
2.No
1. Partially (please specify): ______

28. In your opinion, what are the major challenges associated with cochlear implantation in the UAE? (Open-ended)
